# Selenium-binding protein 1 (SELENBP1) is a marker of mature adipocytes

**DOI:** 10.1016/j.redox.2018.11.004

**Published:** 2018-11-10

**Authors:** Holger Steinbrenner, Mustafa Micoogullari, Ngoc Anh Hoang, Ina Bergheim, Lars-Oliver Klotz, Helmut Sies

**Affiliations:** a Institute of Nutritional Sciences, Nutrigenomics, Friedrich Schiller University Jena, Jena, Germany; b Institute of Biochemistry and Molecular Biology I, Heinrich Heine University Düsseldorf, Düsseldorf, Germany; c Department of Nutritional Sciences, Molecular Nutritional Science, University Vienna, Vienna, Austria; d Leibniz Research Institute for Environmental Medicine, Düsseldorf, Germany

**Keywords:** ACC, acetyl-CoA-carboxylase, DGAT, diacylglycerol O-acyltransferase, GAPDH, glyceraldehyde-3-phosphate dehydrogenase, GPx, glutathione peroxidase, HPRT, hypoxanthine phosphoribosyltransferase, HRP, horseradish peroxidase, IF, immunofluorescence, mTOR, mammalian target of rapamycin, NAFLD, non-alcoholic fatty liver disease, Nox, NADPH oxidase, PARP, poly (ADP-ribose) polymerase, PPAR-γ, peroxisomal proliferator-activated receptor gamma, SELENBP, selenium-binding protein, TNF-α, tumour necrosis factor alpha, VAT, visceral adipose tissue, 3T3-L1, Adipogenesis, Lipid accumulation, NAFLD, Selenium, GPx1

## Abstract

Selenium-binding protein 1 (SELENBP1) has recently been reported to catalyse the oxidation of methanethiol, an organosulfur compound produced by gut microbiota. Two of the reaction products of methanethiol oxidation, hydrogen peroxide and hydrogen sulphide, serve as signalling molecules for cell differentiation. Indeed, colonocyte differentiation has been found to be associated with SELENBP1 induction. Here, we show that SELENBP1 is induced when 3T3-L1 preadipocytes undergo terminal differentiation and maturation to adipocytes. SELENBP1 induction succeeded the up-regulation of known marker proteins of white adipocytes and the intracellular accumulation of lipids. Immunofluorescence microscopy revealed predominant cytoplasmic localisation of SELENBP1 in 3T3-L1 adipocytes, as demonstrated by co-staining with the key lipogenic enzyme, acetyl-CoA-carboxylase (ACC), located in cytosol. In differentiating 3T3-L1 cells, the mTOR inhibitor rapamycin and the pro-inflammatory cytokine tumour necrosis factor alpha (TNF-α) likewise suppressed SELENBP1 induction, adipocyte differentiation and lipid accumulation. However, lipid accumulation *per se* is not linked to SELENBP1 induction, as hepatic SELENBP1 was down-regulated in high fructose-fed mice despite increased lipogenesis in the liver and development of non-alcoholic fatty liver disease (NAFLD). In conclusion, SELENBP1 is a marker of cell differentiation/maturation rather than being linked to lipogenesis/lipid accumulation.

## Highlights

• SELENBP1 is a novel marker of mature adipocytes.
• SELENBP1 was induced during terminal adipocyte differentiation.
• Anti-adipogenic factors suppressed SELENBP1 induction.
• Despite increased lipogenesis, hepatic SELENBP1 was down-regulated in NAFLD mice.

## Graphical abstract

**Figure:**
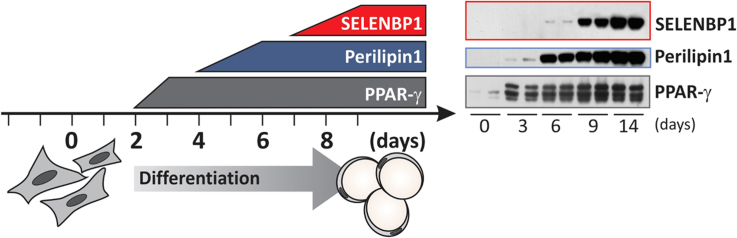

## 1 Introduction

The essential trace element selenium (Se) exerts most of its biological actions through selenocysteine-containing selenoproteins, many of which are enzymes involved in redox regulation and antioxidant protection [1], [2], [3]. In addition, selenium-binding proteins (SELENBPs) including SELENBP1 covalently bind selenite through cysteine residues [4], [5], [6], [7]. SELENBP1 has attracted attention due to its pronounced down-regulation in cancers. For patients, low SELENBP1 levels in tumour tissue are associated with poor prognosis [4], [8], [9], [10], [11].

The physiological role of SELENBP1 has long remained elusive. There is evidence for its involvement in intracellular protein degradation and transport [12], [13]. SELENBP1 is a marker of terminally differentiated epithelial cells in the colon [9], and it may act as tumour suppressor [14]. Recently, an enzymatic activity of SELENBP1 has been discovered: SELENBP1 converts methanethiol, an organosulfur compound derived from gut bacteria, into hydrogen peroxide (H_2_O_2_), hydrogen sulphide (H_2_S) and formaldehyde [15]. Interestingly, H_2_O_2_ and H_2_S are signalling molecules in cell differentiation, including the differentiation of preadipocytes into adipocytes [16], [17]. In the multi-step process of adipogenesis, mesenchymal precursor cells first become committed preadipocytes (adipocyte determination), before undergoing mitotic clonal expansion, terminal differentiation and maturation (adipocyte differentiation) [18]. Both H_2_O_2_ and H_2_S augment adipocyte differentiation and lipid accumulation [19], [20], [21]. Intracellular H_2_O_2_ levels increase during mitotic clonal expansion of preadipocytes, which is counter-balanced through antioxidant enzymes [16], [19], [20], [22]. Also, H_2_S levels increase during adipocyte differentiation, through up-regulation of H_2_S-generating enzymes [21].

This requirement of H_2_O_2_ and H_2_S for adipocyte differentiation [19], [20], [21] together with the roles of SELENBP1 as H_2_O_2_/H_2_S-generating enzyme [15] and as differentiation marker of colonocytes [9] prompted us to study its regulation during adipocyte differentiation of 3T3-L1 cells. This widely used *in vitro* model reflects well the respective processes in primary preadipocytes undergoing adipocyte differentiation [18], [23], [24]. Here, we show that SELENBP1 is a marker of mature adipocytes that is induced during terminal adipocyte differentiation of 3T3-L1 cells.

## 2 Materials and methods

### 2.1 Reagents and antibodies

All chemicals were from Sigma-Aldrich (Munich, Germany) except for tumour necrosis factor alpha (TNF-α) from PeproTech (Hamburg, Germany) and rapamycin from Merck (Darmstadt, Germany). PCR primers were synthesised by ThermoFisher Scientific Life Technologies (Waltham, MA).

The following antibodies were used: anti-SELENBP1 (MBL; Nagoya, Japan); anti-glyceraldehyde-3-phosphate dehydrogenase (GAPDH) (Sigma-Aldrich); anti-acetyl-CoA-carboxylase (ACC), anti-perilipin 1, anti-poly (ADP-ribose) polymerase (PARP), anti-peroxisomal proliferator-activated receptor gamma (PPAR-γ) (Cell Signalling Technology (CST); Beverly, MA); horseradish peroxidase (HRP)-coupled anti-rabbit IgG (Dianova; Hamburg, Germany); HRP-coupled anti-mouse IgG (ThermoFisher Scientific Pierce); Alexa Fluor® 488-coupled anti-rabbit IgG and Alexa Fluor® 594-coupled anti-mouse IgG (CST).

### 2.2 Cell culture

Murine 3T3-L1 cells (CL-173) obtained from the American Type Culture Collection (ATCC) were used between passages 3 and 10 after receipt, and cultured and differentiated into adipocytes as described [23], [25]. Where indicated, the differentiation medium was supplemented with 200 nM selenite, as the Se content in cell culture media is not sufficient to ensure saturated biosynthesis of selenoproteins [26]. Lipid accumulation was assessed by Oil Red O staining as described [25].

### 2.3 Animals

For analysis of SELENBP1 mRNA levels in liver and visceral adipose tissue (VAT) of mice, samples from a previously characterised animal model [27] were used. In brief, female C57BL/6J mice were fed a 30% fructose solution and chow *ad libitum* for 16 weeks to induce non-alcoholic fat liver disease (NAFLD), while controls were fed plain water and chow *ad libitum* [27]. Female mice were chosen, as they are more susceptible to fructose-induced NAFLD than males [28]. All procedures were approved by the local Institutional Animal Care and Use Committee (IACUC).

### 2.4 RNA isolation and real-time RT-PCR

Total RNA was prepared from 3T3-L1 cells using the RNeasy Mini Kit (Qiagen; Hilden, Germany), or from liver and VAT sections of C57BL/6J mice as described [27]. RNA was transcribed into cDNA with SuperScript II reverse transcriptase (ThermoFisher Scientific Life Technologies). For the 3T3-L1 samples, analysis was done in a LightCycler 2.0 qPCR system (Roche; Mannheim, Germany), and PCR amplicons were quantitated by the LightCycler software as described [25]. The mouse samples were analysed in a CFX Connect cycler (Bio-Rad Laboratories; Munich, Germany), and PCR amplicons were quantitated by the CFX Connect software as described [29]. Results were computed as fold changes after normalisation to the mRNA levels of hypoxanthine-guanine phosphoribosyl transferase (HPRT). Primer sequences are listed in Table 1.

**Table 1:** Primers (5′-3′) used for real-time RT-PCR analysis.

**Gene**	**Gene ID**	**Forward primer**	**Reverse primer**
ACC1	NM_133360	ggctcaaactgcaggtatcc	ttgccaatccactcgaaga
DGAT2	NM_026384	tactccaagcccatcacca	ggcatggtacaggtcgatgt
GPX1	NM_008160	ggtggtgctcggtttcccgtg	aattgggctcgaacccgccac
HPRT	NM_013556	tcctcctcagaccgctttt	cctggttcatcatcgctaatc
SELENBP1	NM_009150	tgagcctctgctcgttcc	tggaccacactttgtgcatt

### 2.5 Subcellular fractionation of proteins

3T3-L1 cells were cultured and differentiated into adipocytes until day 14. Cytoplasmic and nuclear fractions were prepared using NE-PER nuclear and cytoplasmic extraction reagents (ThermoFisher Scientific Pierce).

### 2.6 Immunoblotting

Immunoblotting techniques were applied as described [25]. Cells were lysed in ProteoJET Mammalian Cell Lysis Reagent (ThermoFisher Scientific) supplemented with protease inhibitors (Merck). Equal amounts of protein were run on SDS-polyacrylamide gels, and electroblotted onto PVDF membranes (GE Healthcare; Freiburg, Germany). Protein detection was carried out using SuperSignal West Pico (ThermoFisher Scientific Pierce) or SignalFire ECL Reagent (CST) on Hyperfilm ECL (GE Healthcare). For quantitation, films were scanned and analysed using ImageJ software.

### 2.7 Immunofluorescence (IF) microscopy

IF analysis was performed as described [25]. 3T3-L1 cells were grown on glass coverslips and subjected to adipocyte differentiation for 6 days. Cells were co-stained with SELENBP1 and ACC antibodies, followed by incubation with Alexa Fluor®-coupled antibodies. Coverslips were mounted with ProLong Gold anti-fade reagent containing DAPI (CST). Digital images were produced using an Observer D1 microscope (Zeiss; Jena, Germany).

### 2.8 Statistical analysis

Means were calculated from at least three independent *in vitro* experiments and from n = 6 animals/group, respectively. Error bars represent standard error of the mean (S.E.M.). Analysis of statistical significance was done by Student's *t*-test with p < 0.05 considered to be significant.

## 3 Results and discussion

### 3.1 SELENBP1 was induced during terminal adipocyte differentiation, succeeding induction of known biomarkers of white adipocytes

**Fig. 1:**
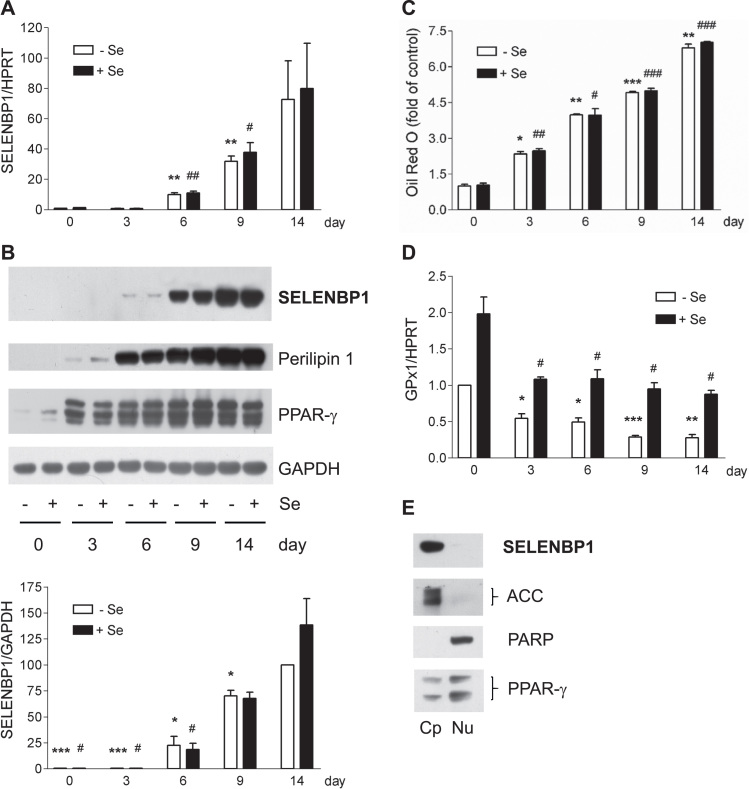
Induction of SELENBP1 in the course of 3T3-L1 adipocyte differentiation. 3T3-L1 cells, cultured with or without 200 nM selenite, underwent adipocyte differentiation for the indicated times (3–14 d). (A) Gene expression of SELENBP1, as analysed by real-time RT-PCR with normalisation against HPRT (n = 4; means ± S.E.M.; **p < 0.01 *vs.* day 0 (-Se); ^#^p < 0.05, ^##^p < 0.01 *vs.* day 0 (+Se)). (B) Protein levels of SELENBP1, as detected by immunoblotting with GAPDH as loading control. Representative immunoblots (upper panel) and densitometric analyses from three independent experiments (lower panel) are shown (n = 3; means ± S.E.M.; *p < 0.05, ***p < 0.001 *vs.* day 14 (-Se); ^#^p < 0.05 *vs.* day 14 (+Se)). Induction of perilipin 1 (marker of lipid accumulation) and PPAR-γ (master regulator of adipocyte differentiation) are shown in additional representative immunoblots. (C) Intracellular lipid accumulation, as measured by Oil Red O staining (n = 3; means ± S.E.M.; *p < 0.05, **p < 0.01, ***p < 0.001 *vs.* day 0 (-Se); ^#^ p < 0.05, ^##^ p < 0.01, ^###^ p < 0.001 *vs.* day 0 (+Se)).). (D) Gene expression of GPx1, as analysed by real-time RT-PCR with normalisation against HPRT (n = 4; means ± S.E.M.; *p < 0.05, **p < 0.01, ***p < 0.001 *vs.* day 0 (-Se); ^#^p < 0.05 *vs.* day 0 (+Se)). (E) Localisation of SELENBP1 in mature adipocytes, as depicted in an immunoblot representative of 3 independent experiments. Subcellular fractions of mature 3T3-L1 adipocytes (day 14) were prepared using the NE-PER kit. For control, the cytoplasmic marker ACC, the nuclear marker poly (ADP-ribose) polymerase (PARP) and the adipocyte transcription factor PPAR-γ are shown. Fractions: Cp (cytoplasm), Nu (nucleus).

SELENBP1 was strongly induced in 3T3-L1 cells undergoing adipocyte differentiation: SELENBP1 mRNA levels began to rise during terminal differentiation (at day 6) and were highest in mature adipocytes (>70-fold induction compared to preadipocytes) at day 14 (Fig. 1A). SELENBP1 protein was not detectable in preadipocytes, and it was induced from day 6 on, with highest levels in mature adipocytes (Fig. 1B). In comparison, protein levels of perilipin 1, a major lipid droplet coat protein and known marker of adipocytes [30], started to rise already at day 3 of differentiation in parallel with intracellular lipid accumulation (Fig. 1B and C). Likewise, mRNA levels of perilipin 1 and adiponectin, a characteristic hormone of white adipocytes, started to rise from day 3 on (data not shown). PPAR-γ, the master regulator of adipocyte differentiation [18], [24], was induced at day 3 and was not further increased in mature adipocytes (Fig. 1B). This indicates that SELENBP1 is a marker of terminal adipocyte differentiation and maturation. Its late induction may make an involvement of H_2_O_2_ or H_2_S derived from the methanethiol oxidase-activity of SELENBP1 in the fine-tuning of adipocyte differentiation improbable, as those signalling messengers are required in early stages of differentiation [19], [20], [21], [22]. A similar pattern of SELENBP1 induction has been observed during differentiation of epithelial cells of the large intestine. Proliferating intestinal cells showed very low SELENBP1 levels that strongly increased in late stages of differentiation [9]. Migration of cells along the colonic crypt-luminal axis was associated with SELENBP1 induction [9].

SELENBP1 binds selenite [4], [5], [6]; however, supplementation with selenite did not further augment SELENBP1 mRNA or protein levels in differentiating adipocytes (Fig. 1A and B). Perilipin 1 and PPAR-γ (Fig. 1B) as well as lipid accumulation in mature adipocytes (Fig. 1C) were also not affected by selenite. Similarly, we and others previously reported that selenite does not influence adipocyte differentiation [25], [31].

SELENBP1 physically and functionally interacts with the H_2_O_2_-reducing selenoenzyme glutathione peroxidase 1 (GPx1), reciprocally interfering with GPx1 expression and activity [10], [32]. Compared to preadipocytes, mature 3T3-L1 adipocytes show lower GPx activity [33]. We hypothesised that SELENBP1 induction is accompanied by GPx1 suppression, and indeed, we observed a down-regulation of GPx1 during adipocyte differentiation (Fig. 1D). As GPx1 biosynthesis depends on Se supply [1], [3], GPx1 mRNA levels were generally higher in the selenite-supplemented cells (Fig. 1D). In contrast to GPx1, other antioxidant selenoproteins are up-regulated during adipocyte differentiation [25], [34], [35]. This is considered as part of the adaptive response to cope with increased production of H_2_O_2_ during mitotic clonal expansion, ensuring cellular redox homeostasis [3], [16].

**Fig. 2:**
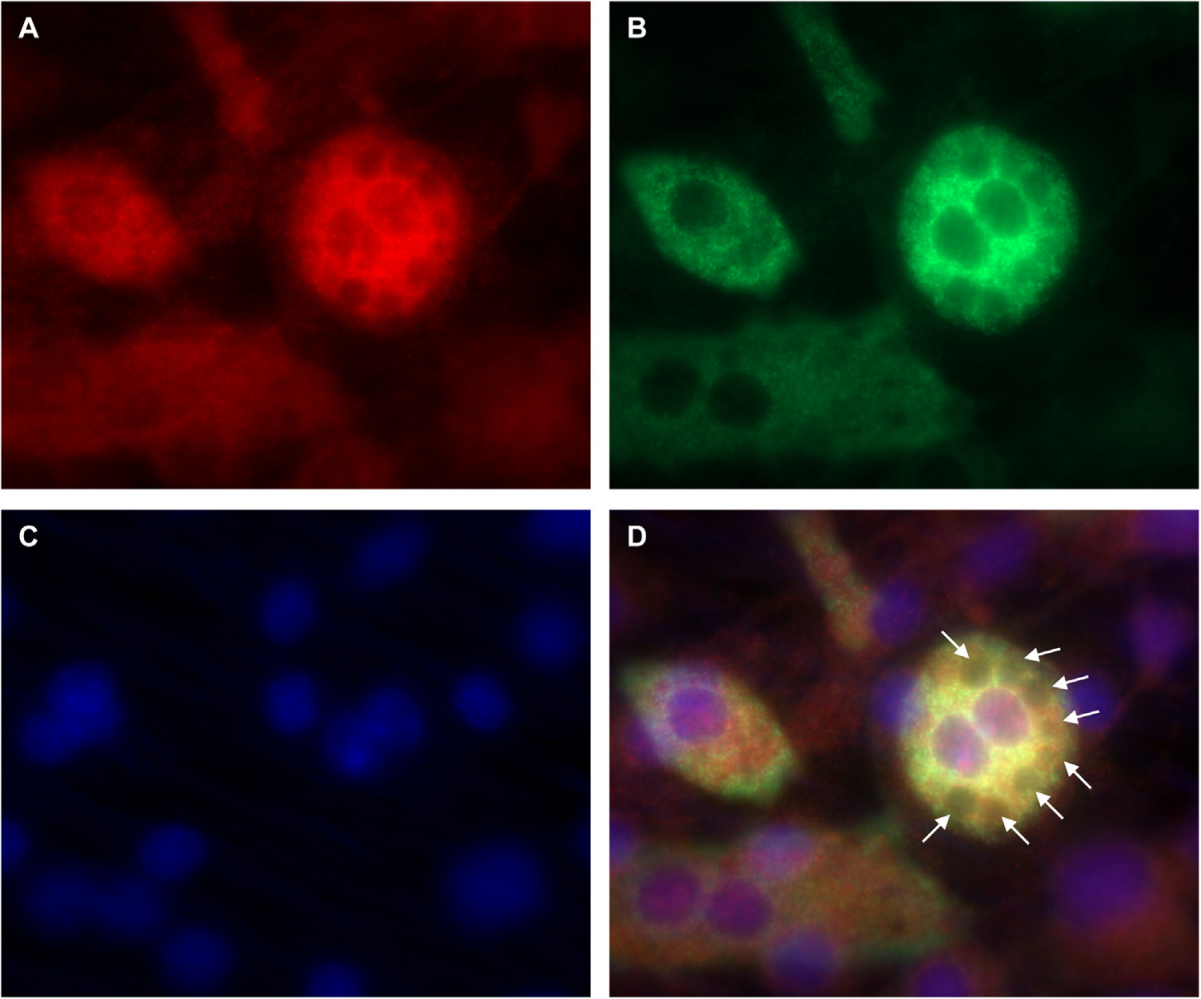
In mature adipocytes, most SELENBP1 co-localises with ACC, the cytosolic key enzyme for fatty acid biosynthesis. 3T3-L1 cells were subjected to adipocyte differentiation for 6 d and subsequent co-immunostaining. (A) SELENBP1 (red), (B) ACC (green), (C) DNA stained with DAPI (blue), (D) merger. The small white arrows in (D) indicate intracellular lipid droplets. The cells are densely packed, as adipocyte differentiation is induced in postconfluent cells undergoing mitotic clonal expansion before terminal differentiation [18], [23], [24]. As previously described [22], the individual cells show unequal rates of differentiation into adipocytes, resulting in a heterogeneous pattern of ACC expression and lipid droplet formation and size.

SELENBP1 resides primarily in the cytosol but it was also detected in smaller amounts within the nucleus and the endoplasmic reticulum of some cells [5], [9], [12]. In mature 3T3-L1 adipocytes, SELENBP1 was present in the cytoplasm, as observed after subcellular fractionation (Fig. 1E). In addition, we co-stained 3T3-L1 adipocytes for SELENBP1 and ACC, which catalyses the rate-limiting step in fatty acid biosynthesis. Only the 3T3-L1 cells with lipid droplets and strong ACC immunoreactivity displayed strong SELENBP1 immunoreactivity (Fig. 2). While the vast majority of SELENBP1 immunoreactivity co-localised with ACC in the cytosol, there was also some punctual SELENBP1 staining in the nucleus of well-differentiated cells (Fig. 2). Our ACC antibody detects both ACC1 and ACC2; however, 3T3-L1 adipocytes can be expected to express almost exclusively (cytosolic) ACC1, being the predominant ACC isoform in lipogenic tissues (including adipose tissue) in mice [36].

### 3.2 Anti-adipogenic factors inhibited SELENBP1 induction in differentiating 3T3-L1 cells

**Fig. 3:**
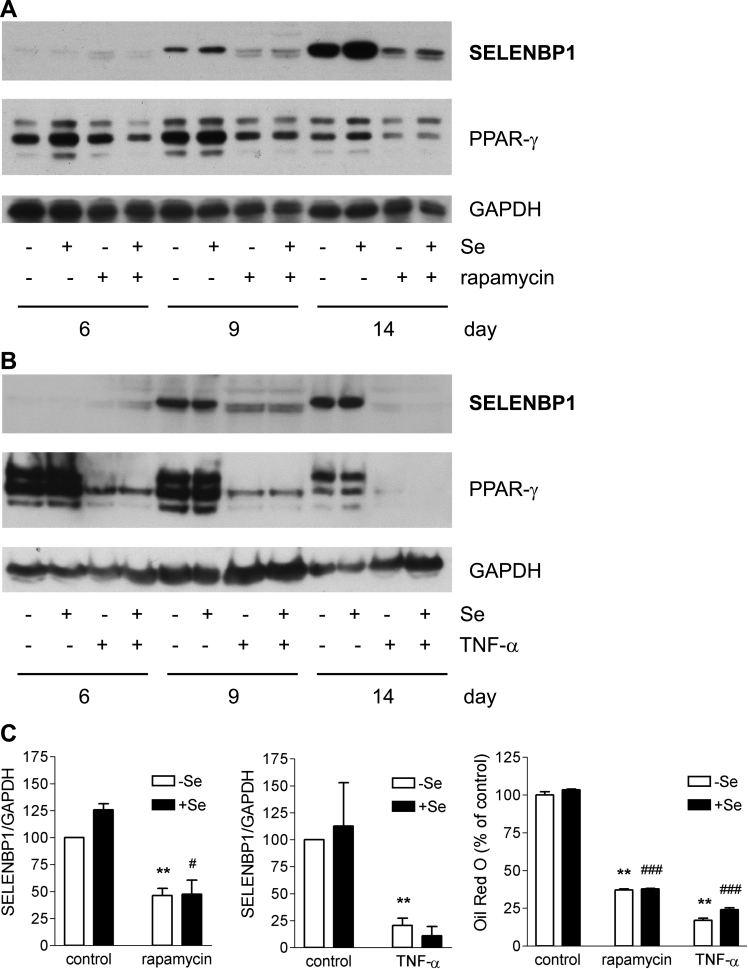
Anti-adipogenic factors suppress SELENBP1 induction in differentiating 3T3-L1 cells. 3T3-L1 cells were subjected to adipocyte differentiation for 6, 9 or 14 d (as indicated) with or without selenite (200 nM) and with or without addition of rapamycin (5 nM) (A) or TNF-α (10 ng/ml) (B). SELENBP1 was detected by immunoblotting. PPAR-γ and GAPDH served as marker for adipocyte differentiation and as loading control, respectively. Immunoblots representative of 3 independent experiments are shown. (C) Relative SELENBP1 protein levels in comparison to intracellular lipid accumulation in 3T3-L1 cells treated with rapamycin or TNF-α for 14 days during adipocyte differentiation. Densitometric analyses of the immunoblots from the experiments with rapamycin (left panel) and TNF-α (middle panel). Lipid accumulation, as measured by Oil Red O staining (right panel) (n = 3; means ± S.E.M.; **p < 0.01 *vs.* untreated (control) mature adipocytes (-Se); ^#^p < 0.05, ^###^p < 0.001 *vs.* untreated (control) mature adipocytes (+Se)).

Development of an adipocyte phenotype is suppressed by treatment with the mammalian target of rapamycin (mTOR) inhibitor rapamycin as well as under pro-inflammatory conditions [37], [38]. 3T3-L1 cells were subjected to the standard adipocyte differentiation protocol in the presence of either rapamycin or TNF-α. As previously reported [25], [37], [38], biosynthesis of the key adipocyte transcription factor PPAR-γ and intracellular lipid accumulation were suppressed under both treatment schemes, with more pronounced anti-adipogenic and anti-lipogenic effects of TNF-α compared to rapamycin (Fig. 3). In parallel, TNF-α as well as rapamycin suppressed SELENBP1 biosynthesis (Fig. 3). Selenite did not modulate the inhibitory effects of rapamycin or TNF-α (Fig. 3).

### 3.3 Induction of hepatic lipogenesis in high fructose-fed mice was accompanied by down-regulation of SELENBP1

Alterations in SELENBP1 expression have been linked to differentiation (in the large intestine) as well as to energy metabolism [9], [39]. In 3T3-L1 cells undergoing adipocyte differentiation, terminal differentiation and lipid accumulation are closely intertwined, being part of the development of an adipocyte phenotype. In order to discriminate between effects related to differentiation or lipid metabolism, we compared SELENBP1 mRNA levels in liver and VAT of mice fed either standard chow and plain water (controls) or standard chow and fructose-enriched water. In this animal model of NAFLD, lipid accumulation takes place without concurrent differentiation, as high fructose consumption induces *de novo* lipogenesis in the liver [40], whereas the histological appearance of the VAT as well as markers of adipogenesis and lipogenesis therein are not altered [41]. As previously demonstrated, the high fructose-fed mice showed pronounced hepatic lipid accumulation and other characteristics of NAFLD [27].

**Fig. 4:**
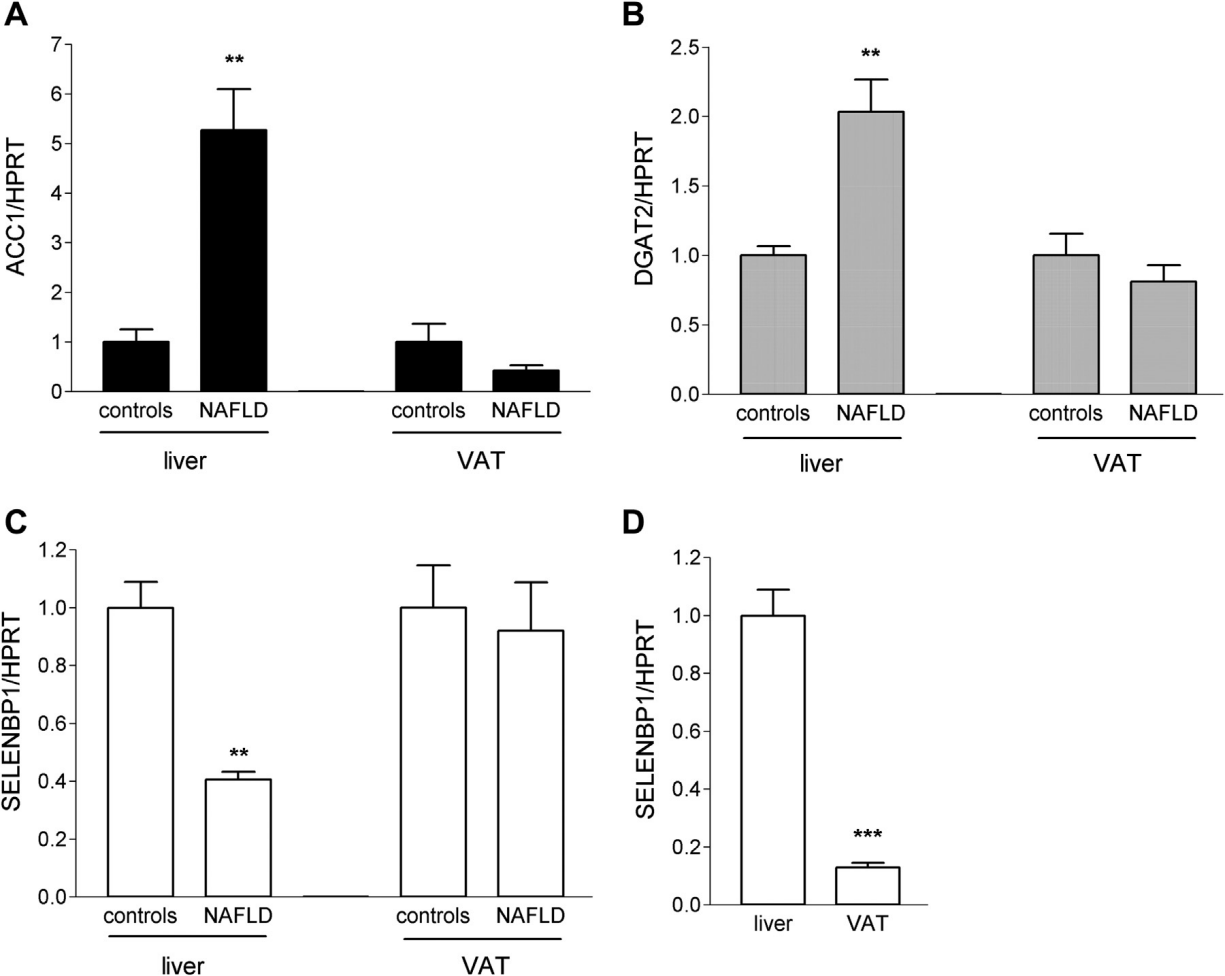
SELENBP1 gene expression is down-regulated in the liver of mice upon induction of NAFLD by a fructose-enriched diet. Female C57BL/6J mice (n = 6/group) were fed either standard chow and plain water (controls) or standard chow and fructose-enriched water (NAFLD) for 16 weeks. Relative ACC1 (A), DGAT2 (B) and SELENBP1 (C). The mRNA levels in liver and VAT of the animals were determined by real-time RT-PCR, with normalisation against HPRT. Liver and VAT mRNA levels, respectively, of the examined target genes in control mice were set as 1 (means ± S.E.M.; **p < 0.01, NAFLD *vs.* control mice). (D) Relative SELENBP1 mRNA levels in VAT of control mice were calculated in relation to respective values in liver set as 1 (means ± S.E.M.; ***p < 0.001, VAT *vs.* liver).

ACC1 was strongly up-regulated (5.3-fold, as compared to controls) in livers of NAFLD mice (Fig. 4A). Moreover, diacylglycerol O-acyltransferase 2 (DGAT2), which catalyses the incorporation of endogenously produced fatty acids into triglycerides [42], was increased 2-fold in livers of NAFLD mice (Fig. 4B). Neither ACC1 (Fig. 4A) nor DGAT2 (Fig. 4B) were significantly altered in VAT of NAFLD mice, as compared to controls. Thus, the differential impact of high fructose consumption on molecular markers of lipogenesis in liver and VAT was also evident in our model. Up-regulation of lipogenesis markers was accompanied by significant down-regulation of SELENBP1 in livers of NAFLD mice, to 40% of the levels in the controls (Fig. 4C).

SELENBP1 has been reported to be ubiquitously expressed, with highest levels in liver, kidneys and intestine [9], [43]. We measured markedly lower SELENBP1 mRNA levels in VAT compared to liver, with levels in VAT at 13% of the values in liver (Fig. 4D). Adipose tissue has been estimated to contain 50–70% mature adipocytes together with stromal preadipocytes, endothelial cells and macrophages [44]. Our data showing induction of SELENBP1 during adipocyte differentiation (Fig. 1) suggest that SELENBP1 is mostly confined to mature adipocytes in the VAT.

## 4 Conclusion

We identified SELENBP1 as a marker of mature adipocytes. Induction of SELENBP1 is linked to cell differentiation/maturation rather than to lipogenesis/lipid accumulation. The function of SELENBP1 in adipocytes remains to be elucidated. As several studies in tumour cells suggest that SELENBP1 counteracts proliferation and dedifferentiation [9], [14], [45], high SELENBP1 levels may support the maintenance of a well-differentiated phenotype in mature adipocytes.
